# APT Attack Detection Scheme Based on CK Sketch and DNS Traffic

**DOI:** 10.3390/s23042217

**Published:** 2023-02-16

**Authors:** Defan Xue, Yaping Chi, Bing Wu, Lun Zhao

**Affiliations:** 1 Beijing Electronic Science and Technology Institute, Beijing 100070, China; 2 School of Telecommunications Engineering, Xidian University, Xi’an 710071, China

**Keywords:** APT attack, sketch, DNS, deep learning, sensor network

## Abstract

In recent years, Advanced Persistent Threat (APT) attacks against sensors have emerged as a prominent security concern. Due to the low level of protection provided by sensors, APT attack organizations are able to develop intrusion schemes that allow them to infiltrate, attack, lurk, spread, and steal information from the target over an extended period of time. Through extensive research on the APT attack process and current defense mechanisms, it has been found that analyzing Domain Name Server (DNS) traffic in the communication control phase is an effective way of detecting APT attacks. However, analyzing APT attacks based on traffic usually involves the detection of a vast amount of DNS traffic, and current data preprocessing methods do not scale down data effectively, leading to low detection efficiency. In previous work, most efforts have been focused on calculating the features of request messages or corresponding messages without considering the association between request messages and corresponding messages. To address these issues, we propose a sketch-based APT attack traffic detection scheme. The scheme leverages the sketch structure to count and compress network traffic, improving the efficiency of APT detection. Our work also analyzes the limitations of traditional sketches in network traffic and proposes an improved sketch scheme. In addition, we propose several effective features for detecting APT attacks. We validate and evaluate our solution using 1,088,280 DNS traffic from a lab network and APT suspicious traffic from netresec and contagio, using eight machine learning models. The experimental results show that for the ExtraTrees model, our solution has a processing time of 0.0638 s and an accuracy of 0.97920, reducing the processing time by approximately 50 times and improving detection accuracy by a small margin compared to a dataset without sketch processing.

## 1. Introduction

With the development of the Internet of Things (IoT), sensors are more commonly used in daily life, such as temperature sensors in cars, sound sensors for controlling lights, and pressure sensors for weighing. Despite the widespread use of sensors, they often lack robust intrusion detection systems compared to traditional hosts and are therefore susceptible to security vulnerabilities, which Advanced Persistent Threat (APT) groups can exploit to launch targeted attacks [1].

In recent years, APT is one of the most representative attacks on the Internet. APT attacks have three main characteristics, which are advanced, persistent, and targeted [2,3,4]. For the characteristic of advanced, attackers can exploit zero-day vulnerabilities to launch attacks that gather a variety of common attacks, such as puddle attacks and phishing emails [5]. Due to the unknown character of zero-day attacks, they are thus able to bypass the current rule-based matching detection system and cannot be defended. In addition, the characteristic of advanced is also reflected in the attack method. Unlike traditional attacks, many APT attacks utilize more stealthy approaches to accomplish their attack purpose. For example, Turla [6] exploits the inherent security flaws of satellite communications to hide the location of C&C servers, MiniDuke tampers with users’ traffic by using Tor to control egress nodes, and APT34 uses DNS tunnels to transmit data. Second, APT attacks generally last for a long time, and once the attacker makes a connection to an intranet machine, he or she tends to engage in long-term lurking or lateral movement to gain greater benefit. Finally, most APT attacks are directed and targeted attacks. The attacker will spend considerable time collecting information on the target based on social engineering, including the target’s interpersonal relationship, commonly used software, network deployment of the target machine, security protection, and so on. This collected information facilitates the attacker to choose the appropriate loading method to penetrate the target machine, thus reducing the cost of the attack and increasing the probability of successful C&C connections.

All APT attacks can be roughly divided into the following five phases: information gathering, embedded intrusion, communication control, lateral penetration, and data returning [7,8]. In the communication control phase, the malware usually uses DNS protocol to communicate with C&C servers, so building detection models for the anomalous DNS traffic characteristics exhibited throughout the process of establishing communication channels between the controlled hosts and C&C servers during APT attacks is an effective approach to detecting APT attacks [9,10,11,12]. However, most of the current approaches for detecting APT attacks based on anomalous DNS traffic have the following two problems: (1) the lack of means to count or reduce the DNS traffic, which leads to low effectiveness of training and testing on massive traffic datasets; (2) most of the DNS traffic features currently proposed for detecting APT attacks focus on the domain name itself, lacking a relationship between requests and accesses and time-dependent features.

Due to the long period of APT attacks and the very covert behavior of each phase, we add features, such as the relationship between DNS requests and DNS responses, access frequency, and time correlation, to those about DNS domain names. As far as the current research is concerned, the huge amount of DNS traffic is a major obstacle to effectively detecting APT attacks [13]. For example, the dataset used to detect APT attacks in the experiments of Li [14] exceeded one billion items. Applying the sketch structure to detect APT attacks based on anomalous DNS traffic can effectively solve the problem.

This article will be organized as four sections:

1. Introduction. Based on the current security situation of sensors, the specific meaning and threats of APT attacks are introduced. This section summarizes the shortcomings of the current research and expounds the main work and organizational structure of this paper.
2. Related work. This part mainly introduces the process of APT attacks and the current mainstream defense measures. Sketch is introduced according to the shortcomings of the above methods, and its existing work in traffic measurement and attack detection is introduced.
3. APT attack detection scheme based on CK sketch and DNS traffic. The proposed scheme for APT attack detection is based on the combination of the characteristics of the APT attack communication control stage and sketch structure. The scheme is analyzed theoretically in depth, and the design ideas and implementation details of the data preprocessing, feature extraction, and the module for counting and compressing DNS traffic with an improved sketch structure, are described in a comprehensive manner.
4. Experience. In the part on the attack detection algorithm, open datasets were used to test its performance. The detection algorithm was evaluated by indicators such as true-positive rate (TPR), precision, accuracy, and processing time, and compared with existing schemes of the same kind to verify its superiority.

The main contributions of this paper can be summarized as follows:

- Sketch structure is used to count and compress DNS traffic containing APT attacks, which improves the efficiency of the approach without reducing the detection accuracy.
- The traditional sketch structure is improved to avoid the error problem caused by the occurrence of hash conflicts in the count min (CM) sketch structure, and the mechanism of adaptively adjusting the number of hash buckets in the sketch structure is added to guarantee the accuracy rate while improving the efficiency of the algorithm for the follow-up.
- The characteristics of APT attack traffic are emphatically analyzed. Based on the characteristics of the DNS domain name, eight features are proposed that can reflect the relationship between DNS request and DNS response, access frequency, and time correlation in APT attacks.
- Combined with eight characteristics of APT attacks, eight machine learning effects are compared. The algorithms include Logistic Regression(LR), KNN, GaussianNB, DecisionTree, Bagging, RandomForest, ExtraTrees, and AdaBoost.

## 2. Related Work

### 2.1. APT Attack Model

**Figure 1:**
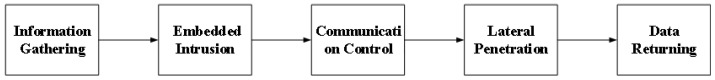
APT attack model.

Different APT attacks have different attack modes but have similar stage characteristics in the attack process. All APT attacks can be roughly divided into the following five stages, which is shown in Figure 1: information gathering, embedded intrusion, communication control, lateral penetration, and data returning.

- Information gathering
  Information gathering is the process by which APT attackers use multiple means to collect various information about the target network [15]. The collected information mainly includes basic information about the network environment, overall architecture, defense system software, and system vulnerabilities of the target system. Based on the collected information, the weak points of the target network are analyzed and targeted attacks are carried out.
- Embedded intrusion
  Embedded intrusion is the stage where the APT attack formally begins, where the APT attacker writes targeted malware to adapt to the target network environment based on the information gathered about the target system network during the intelligence-gathering phase.
- Communication control
  After successfully compromising the target network, the APT attacker continuously invades the internal core network devices based on the control of the internal network devices already gained, which usually requires establishing a command and control communication channel between the C&C server and the target infected host [16,17,18,19]. In this process, the data transmitted between the malware and the C&C server are usually encrypted with SSL, so it is difficult for the target network’s defense system to determine whether the data transmitted in the communication channel are malicious communication with the C&C server or normal communication with a normal external server. However, the communication traffic between malware and C&C servers exhibits a significant difference from normal traffic [20], and APT attackers will employ domain-flux [21] techniques, and infected hosts may try to connect to a large number of domains suspected to be C&C servers.
- Lateral penetration
  Lateral penetration is the stage in which an APT attack spreads to infect hosts. After an APT attacker has compromised one target network host, he continuously elevates his privileges and steals data within the scope of the privileges he has gained. However, APT attack activities are not satisfied with retrieving data on only one machine and need to continuously find new internal network hosts for spreading propagation and infiltrating the core network [22].
- Data Returning
  The data-returning phase sends the discovered data packaged and compressed back to the C&C server to complete the theft of data. The network traffic in the data returning phase is not significantly different from normal network traffic [23], and detection of this phase is difficult to achieve.

### 2.2. Existing APT Detection Methods

Zhao [24] proposed a systematic framework called IDNS, which identifies malicious C&C domains by analyzing DNS traffic and establishes a reputation evaluation system for judging whether the traffic is malicious or not by combining anomalous traffic detection techniques. The features used to evaluate malicious traffic mainly include the characteristics of the DNS domain name itself, lacking features about the time and the relationship between request and response messages.

Cho DX [25] proposed a method to discover suspicious APT based on unknown domain names. The unknown suspected domain names are derived by analyzing DNS logs and traffic, using a similarity comparison of indicators of compromise (IOC) that have appeared in real APT activities and other steps. The difficulty of this method is collecting IOCs of real APT activities.

Fu Yu [26], Shi Yong [27], Yan Guanghua [28], and Xu Congyuan [29], cover most machine learning algorithms, including supervised and unsupervised learning, integrated learning, and deep learning. The accuracy of the classifiers of these improved machine learning algorithms is generally high, but they still need to be validated by datasets constructed in realistic network environments, and the massive amount of traffic data in the experimental part also affects the efficiency of model training. Wang [30] established an APDD framework to collect a large amount of DNS request data and extracted all the features consistent with APT attacks. It uses change vector analysis to build a credit scoring system for DNS domains and outputs the ranking of suspicious domains according to the credit score. The method relies on massive DNS data to determine the credit score of APT attacks; however, the credit score cannot directly determine whether an APT attack has occurred.

Lu Jiazhong [31] compared normal traffic and anomalous traffic by a time-transformed feature approach to distinguish APT attacks, by capturing the signal of malicious traffic, and then inferring the presence of APT attacks, and then using machine learning methods to detect APT attacks in big data.

Wenlin Chu [32] proposed an SVM-based APT attack detection system using the NSL-KDD database to implement method validation, in which principal component analysis (PCA) was used for feature sampling and then compared using multiple machine learning classifiers for classification.

The comparison of the existing APT detection methods is shown in Table 1. Most of the existing detection approaches mentioned above rely on massive amounts of DNS data and lack features about the relationship between DNS requests and responses, which can lead to low detection efficiency and low accuracy for machine learning. The sketch-based APT detection scheme proposed in this paper can significantly reduce the training time of various machine learning models by counting and compressing DNS traffic while guaranteeing the accuracy of the model, which allows defenders to detect APT attacks faster and improve the efficiency of the scheme.

### 2.3. Sketch Structure

With the increasing prevalence of high-speed and non-uniformly distributed network traffic, accurately measuring network properties has become a challenging task. This is due to the sheer volume of network traffic data, which can result in decreased throughput if the statistical measurement method employed has a certain degree of latency.

To address this issue, the field of network traffic statistical analysis has resorted to using sketch structures. This technique involves constructing a hash function to count and compress the network traffic. A sketch structure typically consists of two components: (1) hash function, which calculates the hash value corresponding to the key in the data stream as an index for data compression, aggregation, and storage, and (2) hash bucket, which the sketch structure creator sets to a specific number for storing the required feature values of the data stream.

Sketch structures have found extensive applications in a variety of areas, including traffic analysis, stream size change measurement, large stream detection, and persistent stream measurement. It has been demonstrated that the use of sketch structures can significantly improve the statistical accuracy of network traffic data while simultaneously compressing, aggregating, and storing it [38].

**Figure 2:**
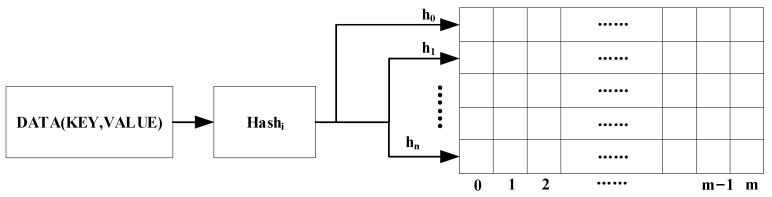
CM sketch structure.

The classical CM sketch, introduced by Cormode G in [39], has been widely utilized in various studies pertaining to network traffic statistics and analysis. This sketch structure is shown in Figure 2. It serves as the foundation for many of its variants and consists of n hash functions and n∗m two-dimensional arrays for compressing and counting network traffic.

When using the CM sketch for network traffic analysis, it is crucial to designate a feature of the traffic, such as the DNS domain name or the Source IP, as the key and the rest of the features as the values to be counted. The location of the network is calculated using the n hash functions as specified in Equation (1).

(1) $$loc_{i}=H_{i}\left(key\right),$$

Each network traffic is mapped to n hash buckets, and then the value in the network is added to the value in the hash bucket.

## 3. Research Design

In this section, we will describe the process of the proposed scheme in this paper and describe each part of the framework in detail in the subsections. This scheme is based on sketch structure and detects whether APT attacks occur by analyzing DNS traffic. It can report to the security officers quickly when it finds suspicious domains, and it can help the defenders discover APT attacks faster.

### 3.1. Overview of the Approach

**Figure 3:**
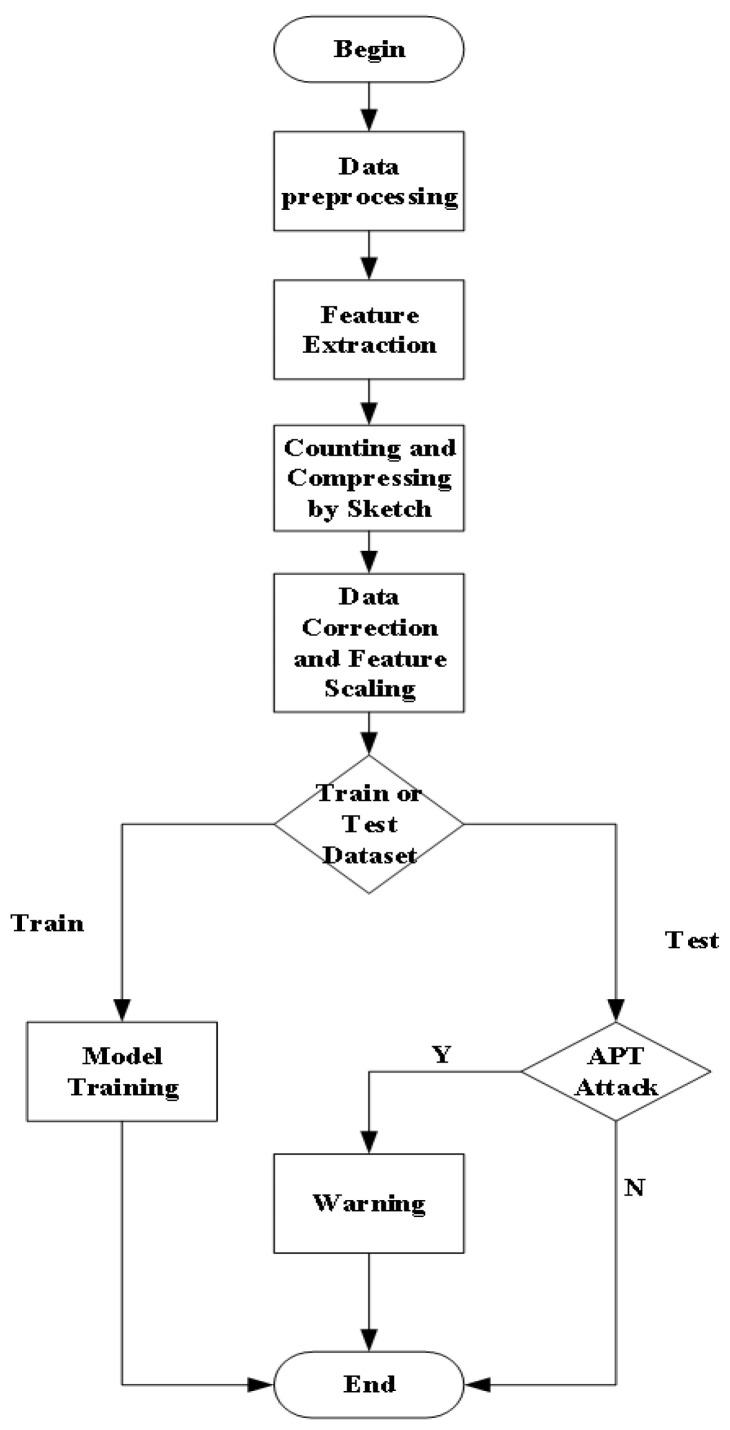
Overview of the approach.

The running flow of the APT attack detection scheme proposed in this paper based on Cuckoo (CK) sketch and DNS traffic is shown in Figure 3. The first operation of the original traffic is data preprocessing. Since the number of original datasets is very large, this paper reduced the dataset from the three perspectives of request type filtering, white list filtering, and access frequency filtering. Furthermore, we extracted the second-level domain name to facilitate the subsequent feature extraction.

Next, we selected the domain names in the DNS traffic as the key, and for the communication control phase of the APT attack, we selected eight features as parameters to outline the network state statistics, the specific types and characteristics of these features will be described in Section 3.3. The DNS traffic is counted and compressed in the form of a sketch structure, where each hash bucket in the sketch will output a data sample of dimension 8, and these hash buckets are themselves linked to a DNS domain name. Thus, the abnormal network state and traffic caused by the abnormal source DNS domain name can be demonstrated by the variety of features. The above features become the prototype of the training and testing feature set of the machine learning model in the subsequent steps.

After the original dataset has been counted and compressed by sketch structure, several types of preprocessing operations are required, mainly including outlier removal, missing value filling, feature scaling, etc., to improve the dataset and enhance the classification effect and efficiency of the machine learning model.

Finally, the dataset after the above processing is the dataset to be classified. In order to build the machine learning model and test its attack detection effect, we divided the dataset into a training set and test set in the ratio of three to one. The training set is marked as normal and abnormal according to the mapping relationship between the DNS domain name and grid serial number in the sketch in advance. To solve the dichotomous classification problem proposed in the last step of the algorithm, we chose eight typical classifiers for attack detection, trained and tested these eight models, recorded the results, evaluated the respective characteristics and learning effects, and selected the optimal detection model.

### 3.2. Data Preprocessing

Malicious DNS traffic from APT attacks is mixed in with normal traffic, and DNS traffic from internal network hosts can be very large, resulting in a very large size of raw data collected in the end. Therefore, it is necessary to preprocess the data, reduce the amount of data processed by the detection model, and ensure that the original data has the same characteristics compared with the processed data. While improving the detection efficiency, the detection results are not affected.

**Figure 4:**
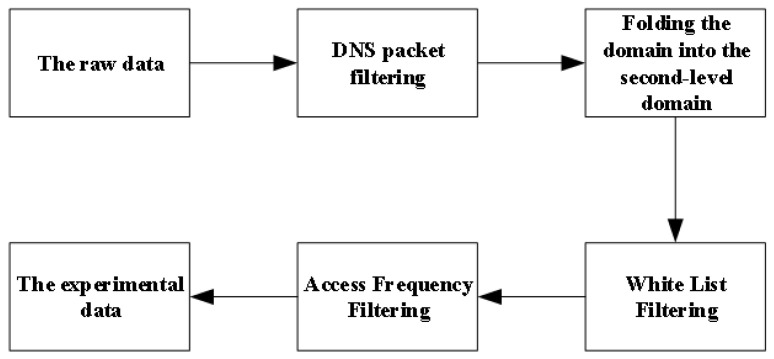
Data preprocessing process.

The data preprocessing consists of four parts in total, DNS packet filtering, folding the domain into the second-level domain, white list filtering, and access frequency filtering. The specific algorithm flow is shown in Figure 4.

- DNS packet filtering

**Figure 5:**
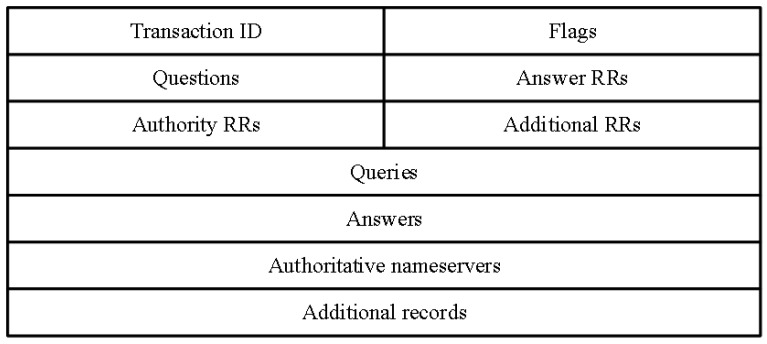
DNS packet format.

As can be seen from Figure 5, both the query and response data areas of DNS packets contain two fields, query type and query class. In this paper, only DNS packets with query class IN and query type A are processed. The query class is IN, which indicates the Internet category; the query type A indicates the IP direction of the web server, and the type A (address) is used to specify the IPv4 address corresponding to the domain name. The DNS packets of other query types are not processed, and only the DNS packets requesting IPv4 addresses are retained for the first filtering of the original data.

- Folding the domain into the second-level domain

Since second-level domains are of higher value for identifying apt malicious traffic, all domains are converted into second-level ones, such as www.zhihu.coa and wenda.zhihu.com, which will be transformed into zhihu.com.

- White List Filtering

According to the characteristics associated with APT attacks, APT attackers expect to be able to control the infected host for a long time, so they need to send commands remotely through a C&C server. Using a server with a popular domain name as a C&C server can achieve a good stealth effect, but it requires a great cost. An APT attacker has to compromise a server with a popular domain name that has a well-developed defense system installed, which undoubtedly increases the risk of exposure and is easy to detect by network defenders. Therefore, it is generally believed that popular domain names are not likely to be used for APT attacks. With such a premise, this paper proposes a white list filtering detection algorithm. The top 10,000 domain websites can be obtained from the Alexa website, and the domain white list is constructed based on the obtained domain names. If the resolved domain name belongs to the white list, no further processing is performed for the domain name and the current DNS data message is discarded directly.

- Access frequency filtering

APT attacks are a low-frequency attack mode in which the malware in the internal network host wants to initiate connection requests with external C&C servers by sending DNS request messages, which are not very frequent. The number of DNS requests sent in a day must be below a certain threshold, thus greatly reducing the risk of detection by the defense system. In this paper, the threshold is set to 100 times/day, and a domain name that is accessed more than 100 times in a day is considered normal, and the domain name is removed from the candidate domain name results.

### 3.3. Feature Extraction

1. Domain Length
  In APT attacks, attackers usually use the domain generation algorithm (DGA) algorithm to generate a series of domain names, which are generally longer in length. There are two reasons for this: first, the shorter domain names may have already been registered by organizations or individuals, and the domain names generated by the algorithm may conflict with the already registered algorithm, resulting in the failure of the C&C server domain name; then, the attacker uses registered second-level domain names to reduce maintenance costs, and the overall length of the domain name is longer. Due to such a process of constructing domain names, this paper argues that the length of the domain name can be used to determine whether it is a malicious domain name.
2. Number of Visits to the Domain
  Whether it is the initiation of communication control or the lateral penetration phase, in order to avoid detection, the attacker acts very stealthily and rarely initiates connections with external servers. So, the fewer the number of visits to the domain to be detected the more suspicious the domain is.
3. Access Period
  Under normal circumstances, the time of the host accessing the domain name should show a certain regularity related to people’s work and rest. APT attackers, in order to improve the stealth of malware connections, usually initiate DNS requests when people are sleeping, thus reducing the chance of being detected by humans. It is reasonable to assume that DNS access requests during the period of 0 a.m. to 6 a.m. each day are suspicious.
4. Digital Feature
  According to several malicious domain names with numbers that have been found, malicious domain names usually change some letters to numbers or add some numbers to the regular domain names. The domain names generated by the DGA algorithm based on the domain-flux technology generally contain numbers. APT attackers also deliberately alter domain names to pretend to be normal ones, for example, the letter “o” might be replaced with a 0, and the letter “l” with a 1. Calculate the proportion of digits based on whether the domain name contains digits. As for the numerical feature of the domain name, focus on the domain name that contains digits 0 or 1.
5. Packet Length Characteristics
  According to the characteristics of APT attack traffic analyzed above, the downstream traffic is generally the control commands sent by APT attackers, while the upstream traffic is the result of the information returned by the controlled host after the execution of the control commands. This is different from normal DNS traffic. In this section, the average length of upstream and downstream packets is calculated respectively as the feature of packet length.
6. Domain Name Request and Response Interval
  In some APT attacks, the attacker-controlled DNS servers are not guaranteed to be online for a long time to increase the stealthiness, so the malware does not receive the IP address of the C&C server when it initiates a request message for the C&C server domain name. Based on such a situation, this paper proposes the time interval between DNS domain name response and request as a feature for the following reasons: in APT attacks, the DNS resolution process for the C&C server domain name is unusual, and this feature can reflect the complete resolution process; the frequency of malware-initiated DNS requests should be below a certain threshold, and the malicious domain name is not in the corresponding cache of the DNS server, and it takes more time to query the IP address corresponding to the domain name. If the time interval between the response and the request is too large, the domain name can be considered suspicious.
7. Time to Live (TTL)
  TTL is the retention time of domain name resolution records in DNS servers, increasing the TTL value can accelerate the domain name resolution time, and decreasing the TTL value can accelerate the effective time of domain name resolution. The default TTL is currently given as 3600 s, and the value can be adjusted by itself as needed. Leyla [40] summarizes the DNS features used until 2018 and mentions the body of literature about TTL as a feature for detection. The TTL distribution of APT traffic has variability from normal traffic, so the TTL value is added to the classification vector as a feature dimension. The TTL values of normal traffic are mostly concentrated below 1000, but there are several values above 1000; the TTL values in the DNS dataset of APT are distributed at both ends, with one end distributed at 60 and below, and the other end distributed at around 100.
8. Domain Access Interval
  Under normal circumstances, the time interval between two visits to the same domain by internal hosts is not too small. The attacker has a long period of latency during the lateral penetration phase, in which the infected hosts rarely visit external servers to hide their behavior, so the longer the interval between domain accesses, the more suspicious the domain is.

Among the above eight features, features 1, 4, and 5 do not need to be accumulated and can be calculated directly using domain names; features 2 and 3 are accumulated using counting; features 6, 7, and 8 are calculated and measured in the form of entropy.

### 3.4. Count and Compress DNS Traffic with an Improved Sketch Structure

In Section 2, we discussed sketch structure, an important tool in network traffic computation which has gained attention in the field of network attack detection with the advantage of small space and time overhead. Traditional sketch structures, such as the Count sketch and CM sketch, are employed for detecting abnormal network traffic by setting the number of hash buckets to a predetermined value. This is due to the unpredictable nature of network traffic during analysis. However, this approach can result in hash collisions when the network traffic is high and lead to an inefficient utilization of memory and computational resources when the traffic is low.

To address these challenges, the present study proposes an improved CK hash sketch structure. This structure consists of a multi-layered sketch architecture that employs multiple hash functions and hash buckets. The creation of the different sketch layers is guided by the principle of cuckoo hash, enabling the resolution of hash conflicts.

The proposed CK sketch structure dynamically adjusts the number of hash functions and hash buckets based on the size of the network traffic data, thereby improving throughput and reducing the occurrence of hash collisions during statistical analysis. This results in a more efficient utilization of resources and improved accuracy of network traffic analysis.

#### 3.4.1. Data Structure

The Bloom filter [41] is a data structure used to represent a collection or determine whether an element belongs to a collection. This data structure is a simple and efficient storage structure. It was used in database at first, and has been widely used in network in recent years [42].

**Figure 6:**
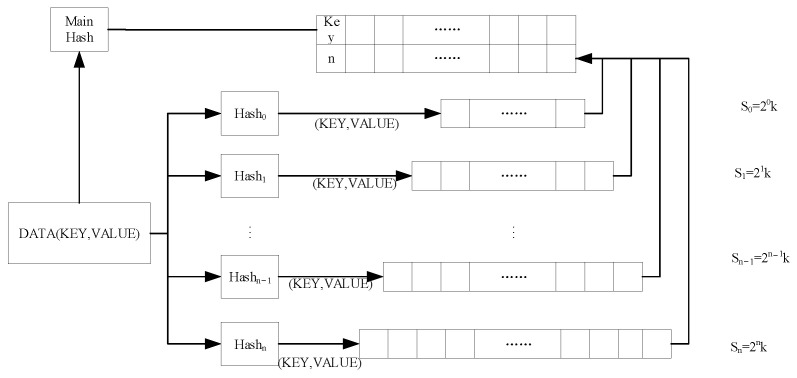
CK sketch data structure.

To attain optimal analysis of network traffic statistics, the CK sketch data structure proposed in this study is shown in Figure 6. This data structure encompasses multiple layers of hash buckets and Bloom filters, which serve to record the storage location of network traffic.

1. Hash bucket: The incoming network traffic is comprised of (Key, Value) pairs, where the key is represented as the DNS domain name, and the value is a set of eight feature values selected to describe APT attacks, as outlined in Section 3.3. The $H_{n}$ function of each layer computes the key present in the network traffic and maps the relevant (Key, Value) pair to the appropriate hash bucket, based on the calculated value.
  (2) $$loc=H_{n}\left(key\right),$$
  The outcome of the hash function operation varies between each layer, and to comprehensively account for the heterogeneity of network traffic, the number of hash buckets in layer *n* + 1 is double that of layer *n*.
  (3) $$S_{n+1}=2S_{n},$$
2. Bloom filter: This structure encompasses the main hash function and a two-dimensional vector. The main hash function is employed to map the network traffic into the two-dimensional vector, where the key is recorded in the first line of the corresponding position in the mapping. Upon completion of data insertion, the number of layers in which the data are located will be recorded in the second row of the corresponding position in the mapping.

#### 3.4.2. Counting and Compressing

**Figure 7:**
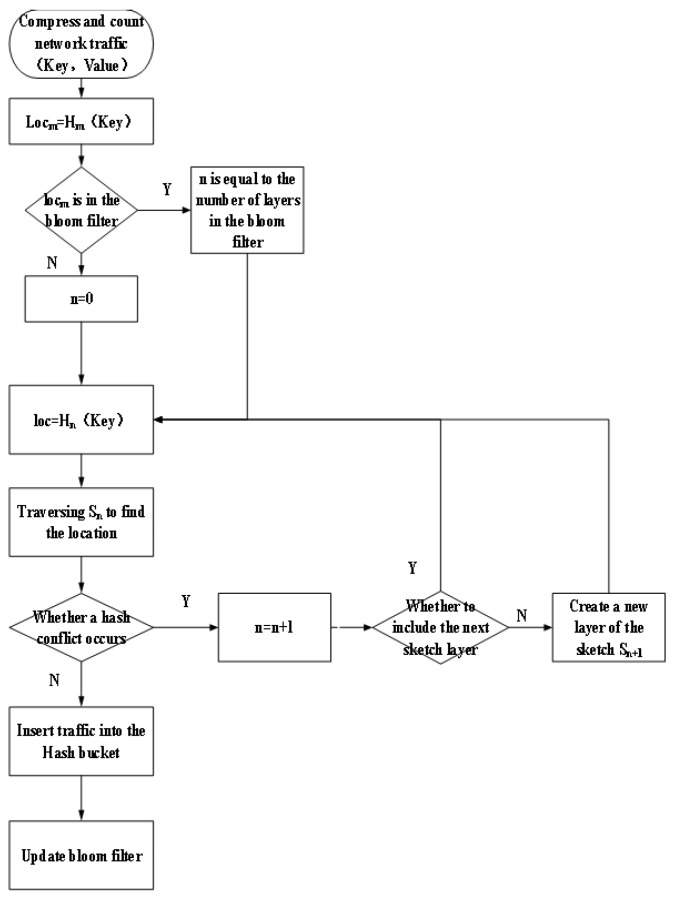
Compress and count flow chart.

The process of compressing the statistical DNS traffic (Key, Value) is shown in Figure 7 and consists of the following five steps, where the key is the DNS domain name and the value is the eight features used to describe the APT attack in Section 3.3.

**Figure 8:**
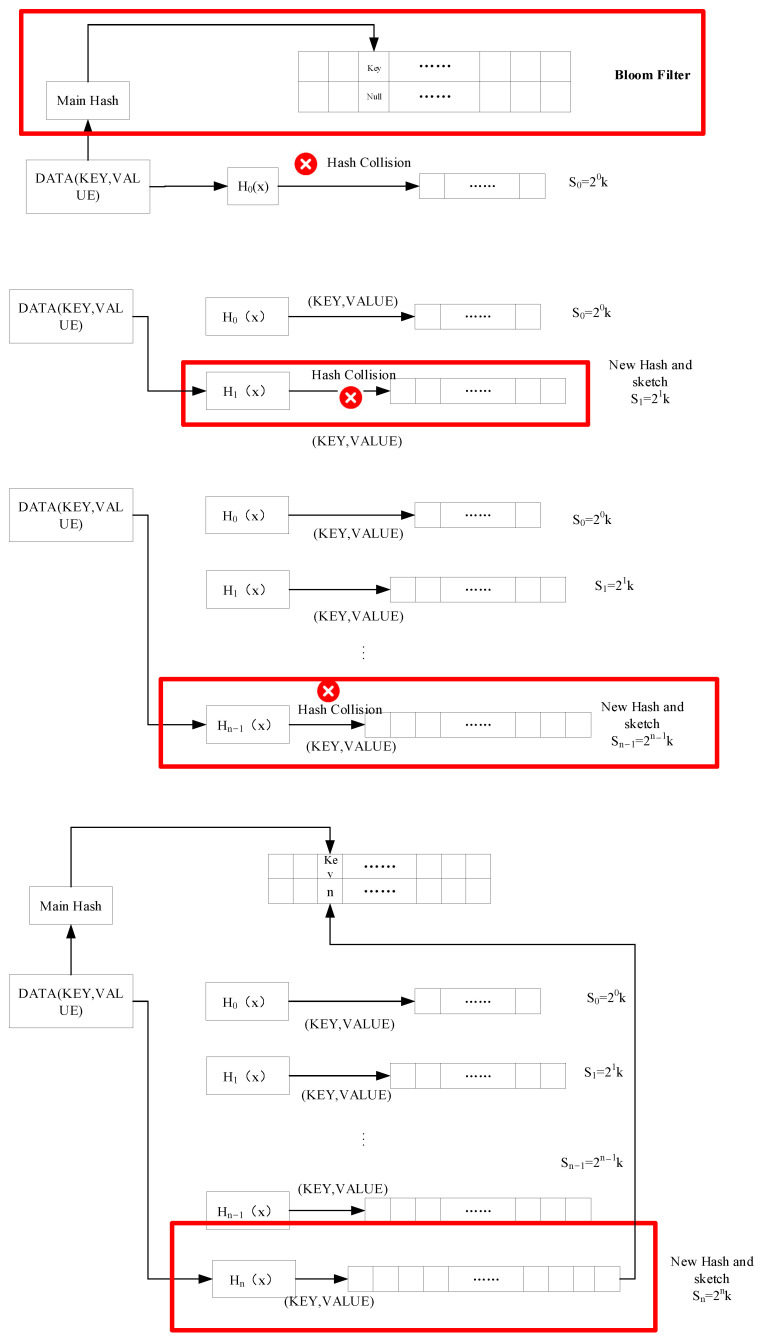
Example of hash collision.

1. Determine whether the current DNS traffic is already located in the hash bucket. The key in the network traffic (Key, Value) is calculated by the main hash function through Equation (4) to obtain the $Loc_{m}$.
  (4) $$Loc_{m}=H_{m}\left(key\right),$$
  According to whether the $Loc_{m}$ position in the Bloom filter already contains elements, corresponding to the following three cases.
  - There are no data in this position, then the element is added to the $Loc_{m}$ position in the Bloom filter, and the element is stored in the multi-layer hash bucket from the first layer.
  - There are data in this location but the corresponding key is different from the corresponding key of the current DNS traffic, the element will be stored in the hash bucket of the multi-layer starting from the first layer.
  - There are data in this location and the corresponding key is the same as the corresponding key of the current network traffic, the element will be stored in the corresponding layer of the multi-layer hash bucket directly according to the number of layers in the Bloom filter.
2. Calculate the location of the hash bucket where the DNS traffic is stored. Put the network traffic (Key, Value) into the nth layer according to step 1 and Equation (5) to determine the location where the DNS traffic is stored in the nth layer.
  (5) $$Loc_{n}=H_{n}\left(key\right),$$
3. Determine whether there is a hash conflict. If the $Loc_{n}$ position in the hash bucket does not contain any data, then there is no hash conflict. If there are already data in it and the data are the same as the data to be inserted, then there is no hash conflict, otherwise, there is a hash conflict.
4. Count and compress network traffic (Key, Value). If there is no hash conflict, the key in the network traffic is recorded, and the value is added to the value in the hash bucket. If a hash conflict occurs, create a different hash function $H_{n+1}$ and a layer of hash bucket $S_{n+1}$, where the number of hash buckets in $S_{n+1}$ is twice the number of $S_{n}$. The hash function of layer *n* + 1 is used to calculate Formula (6) to obtain the location of layer *n* + 1 traffic, and insert this DNS traffic into the layer *n* + 1 hash bucket according to the above steps. Finally, the number of layers where the traffic resides is recorded in Bloom filter. Figure 8 shows an example of step 4 counting and compressing DNS traffic (Key, Value) with hash conflict. At this time, after main hash calculation, the Bloom filter does not contain the number of layers of the traffic. Therefore, counting and compressing network traffic started from the first layer of the CK sketch.
  (6) $$Loc_{n+1}=H_{n+1}\left(key\right),$$
5. Record the number of hash bucket layers where the DNS traffic (Key, Value) is located. After counting and compressing, the number of layers where the DNS traffic is located will be recorded in the Bloom filter.

## 4. Experiments

### 4.1. Data Set Description

The experimental dataset consists of two parts of data, which are APT malicious DNS traffic provided by netresec and contagio and normal DNS data from the lab. The malicious traffic dataset contains 15 different types of malicious network traffic from the sources shown in Table 1 below. The secure network traffic comes from a week of DNS traffic within the lab and contains 1,088,280 DNS records. The above malicious data are filtered, and the DNS traffic part of them is selected and combined with the DNS traffic of the lab to obtain the experimental dataset, and the distribution of attributes is shown in Table 2.

### 4.2. Experimental Environment

To verify the performance of the algorithm proposed in this section, the test program was deployed on a host computer with Intel(R) Core(TM) i7-7700HQ CPU @ 2.80 GHz 2.81 GHz, 16G RAM, Nvidia GTX 1060 GPU, Win10 OS, Python 3.6 version.

### 4.3. Metrics

True Positive (*TP*), indicating samples that are malicious traffic and are correctly detected as attack traffic.

True Negative (*TN*), indicating samples that are actually normal traffic and are correctly detected as normal traffic.

False Positive (*FP*), indicating samples that are normal traffic but are incorrectly detected as attack traffic.

False Negative (*FN*), indicating samples that are malicious traffic but are incorrectly detected as normal traffic.

1. TPR
  True Positive Rate (*TPR*) means the proportion of true positive samples to all positive samples in the current positive sample, which is the accuracy of the measurement. The accuracy of the measurement is also known as sensitivity. It is expressed by Equation (7).
  (7) $$TPR=\frac{TP}{TP+FN},$$
2. Precision
  Precision means the proportion of samples that are positive and correctly classified as positive to all samples classified as positive. It is expressed by Equation (8).
  (8) $$\mathrm{Precision}=\frac{TP}{TP+FP},$$
3. Accuracy
  Accuracy means the percentage of all samples that are correctly detected. It is expressed by Equation (9).
  (9) $$\mathrm{Accurary}=\frac{TP+TN}{TP+FP+TN+FN},$$
4. F-measure
  F-measure, also known as F1-Score, combines the precision and recall considerations of detection accuracy and is the summed average of the two, calculated using Equation (10).
  (10) $$\mathrm{F-measure}=\frac{2\times Precision\times Recall}{Precision+Recall},$$
5. Throughput
  It is the number of successfully processed data per unit of time. The experimental part uses the number of packets successfully deposited into the sketch structure per second as the throughput. Higher throughput means faster and shorter processing time for network traffic and better performance.
6. Processing time
  Our scheme first performs feature extraction and summary statistics through CK sketch, and then includes machine learning model training and prediction after preprocessing. The data preprocessing and resampling parts contain a lot of manual offline operations, and the running time is difficult to count, so we only focus on the time consumed by the construction of the machine learning part of the classification model and test set prediction.

### 4.4. Model Training and Results

#### 4.4.1. APT Detection Results

The selected dataset is compressed and counted using the CK sketch structure after the preprocessing operation. The eight features illustrated in Section 3.3 are selected and eight classical machine learning models are used for training and testing. The results are shown in Table 3.

From Table 4, we can see that the six models of KNN, DecisionTree, bagging, RandomForest, ExtraTrees, and AdaBoost can achieve an accuracy rate of 0.96 or higher, among which DecisionTree, ExtraTrees, and RandomForest consume less time in training and testing. The time consumption is lower, which proves the effectiveness of our proposed scheme. Overall, DecisionTree achieves both high detection accuracy and detection efficiency.

#### 4.4.2. Testing Duration Comparison

**Figure 9:**
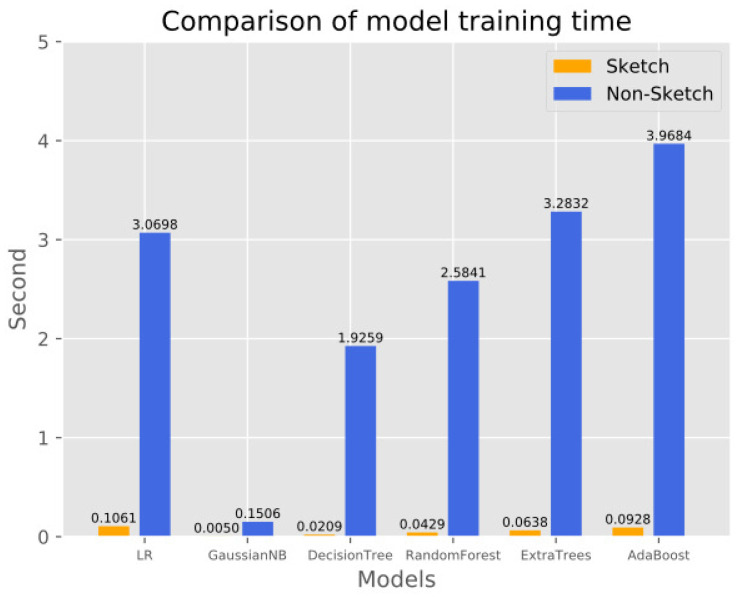
Comparison of training time of six models before and after sketch processing.

**Figure 10:**
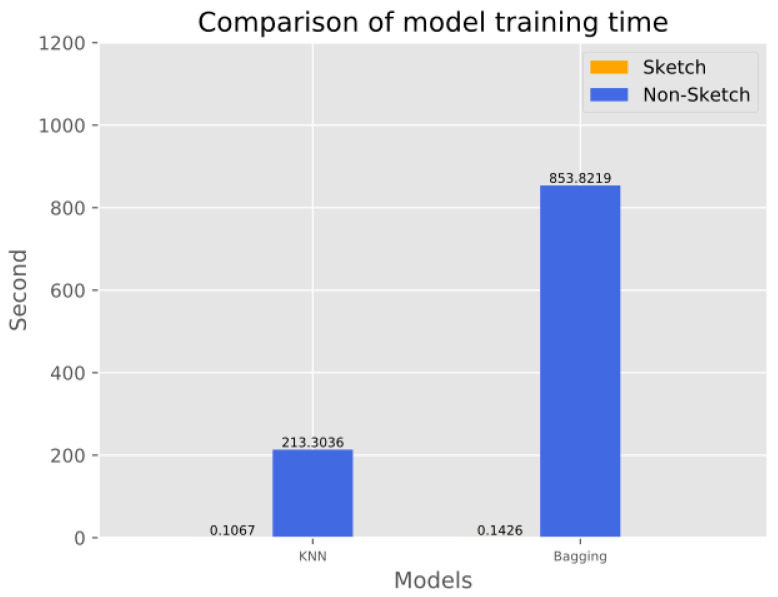
Comparison of the training time of two models before and after sketch processing.

This experiment compares the effect of sketch structure on the training time of various machine learning models in the proposed scheme. In the experiments, we used the same dataset to train all the models, record the time required to finish training the models, and then used the dataset counted and compressed by the sketch structure to train the models and record the time required. The experimental results are shown in Figure 9 and Figure 10, where the horizontal coordinates represent the different machine learning models and the vertical coordinates represent the time spent on model training. The experimental results show that the traffic data, after being statistically compressed by sketch, can significantly reduce the training time of all machine learning models, among which the KNN and Bagging models reduce the time most significantly. The reason is that after the data are compressed by sketch, the traffic from the same DNS domain will be counted and compressed into a hash bucket, which makes the number of training samples drop significantly and saves a lot of time.

#### 4.4.3. Testing Accuracy Comparison

**Figure 11:**
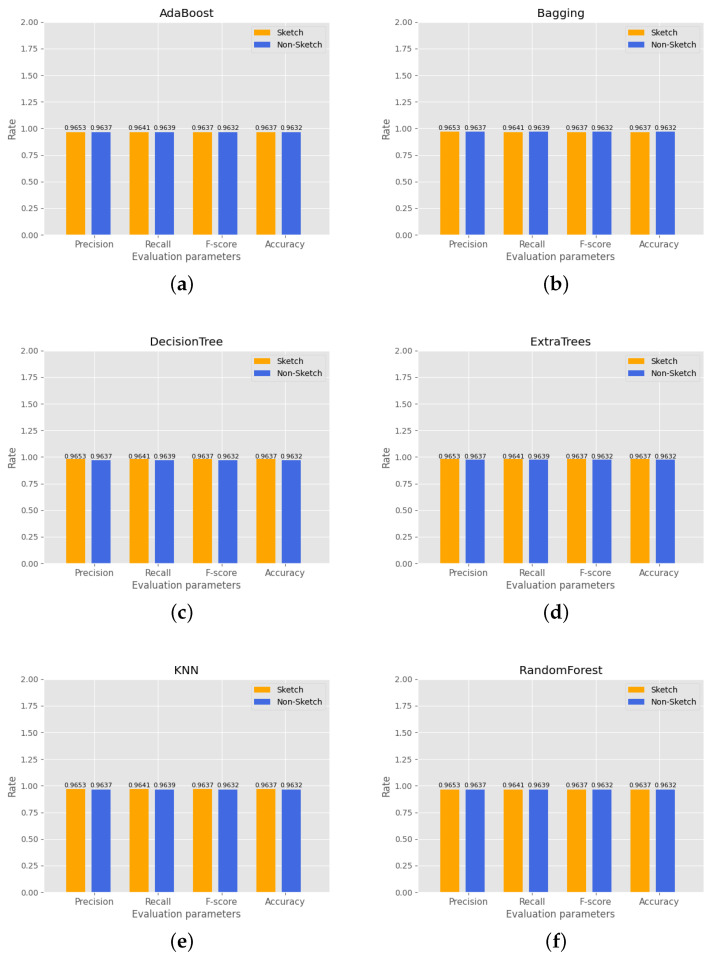
Comparison of model training accuracy before and after sketch processing: (**a**) Adaboost accuracy; (**b**) Bagging accuracy, (**c**) DecisionTree accuracy; (**d**) ExtraTrees accuracy; (**e**) Definitions accuracy; (**f**) RandomForest accuracy.

This experiment compares the accuracy difference between six machine learning models trained and detected using the original dataset and the dataset compressed and counted by CK sketch. The experimental results are shown in Figure 11, where the horizontal coordinates represent the four metrics used to measure the detection accuracy of the models and the vertical coordinates represent the accuracy values. The experiments show that, except for RandomForest, the other five machine learning models exhibit higher accuracy when trained and detected with the CK sketch structure.

#### 4.4.4. Throughput Comparison

**Figure 12:**
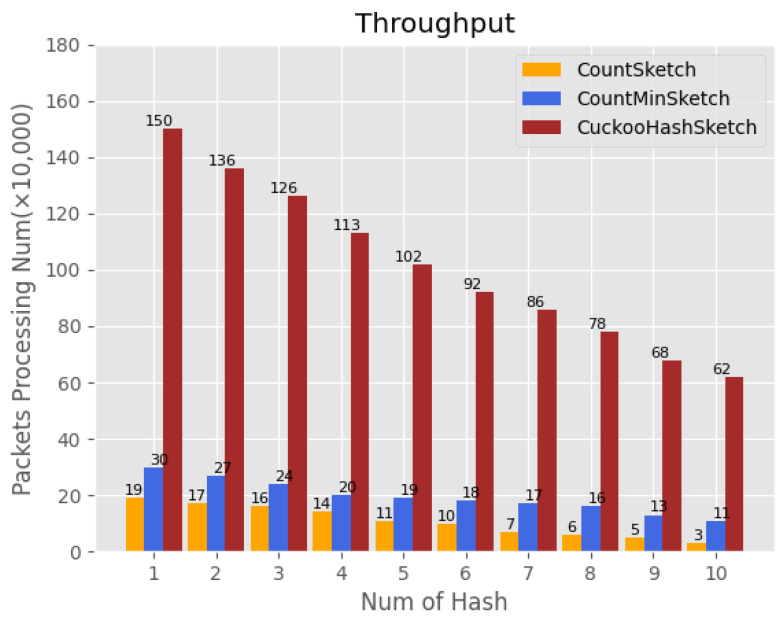
Throughput comparison.

In this experiment, the objective was to compare the processing efficiency of network traffic among different sketch structures. The experiment evaluated the throughput of CK sketch, CM sketch, and Count sketch when compressing and counting network traffic. The results of the experiment are depicted in Figure 12, where the horizontal axis represents the number of hash functions present in each sketch structure and the vertical axis depicts the number of packets processed per second. The findings reveal that as the number of hash functions increases, the processing efficiency of all methods for network traffic data decreases. However, CK sketch exhibits a considerable advantage over the other methods in processing network traffic, owing to the fact that, unlike CM sketch and Count sketch, CK sketch only requires multiple hash calculations in case of hash conflict and thus avoids excessive hash operations and saves a considerable amount of time.

### 4.5. The Conclusion of Experiment

The experimental results show that the scheme is effective in detecting APT attacks and drastically reduces the time for training multiple machine learning models. We compare the performance of CK sketch with two other sketch structures in terms of throughput and based on the results we can see that the throughput of CK sketch is much larger than the other two sketch structures. Using the sketch structure to count and compress massive DNS traffic can effectively improve the efficiency of detecting APT attacks and reduce losses.

### 4.6. Comparison with Previous Work

In the scheme proposed by Yan Guanghua, the author used eight DNS traffic features combined with deep learning to judge APT attacks. However, these eight features lack the consideration of APT attack access time and TLL value in DNS traffic packets, resulting in the failure to accurately detect APT attacks with time rules. Therefore, on the basis of some of the above eight features, this paper adds access time period, packet length, TTL, and other features, so as to obtain a more accurate detection effect. In the part of data preprocessing, the author mainly uses the means of domain name ranking and access times to screen the initial data, and this method has a certain effect. However, the amount of data cannot be reduced effectively, which leads to the training time of the model being too long and the decrease in detection efficiency. Therefore, this paper introduces the CK sketch structure to compress and count the DNS traffic while guaranteeing the accuracy, and significantly reduce the time for model training. However, due to data privacy, the above authors’ datasets were not available. In the comparative experiment, the dataset in Section 4.1 was used to compare the accuracy and model training time of this paper and Yan Guanghua with the same amount of data. The experimental results are shown in Table 5. We find that the introduction of new features in this paper can improve the accuracy of APT attack detection to some extent. More importantly, the use of the CK sketch structure in this paper greatly reduces the time of model training, because the CK sketch structure uses statistics and compresses the data used for model training.

### 4.7. Discussion of Limitations

Since CK sketch uses domain names as the key to compress and count DNS traffic, the diversity of domain names in datasets will affect the time superiority of the scheme proposed in this paper. Therefore, we filter the dataset in Section 4.1 and tested only one DNS traffic with the same domain name. The experimental results are shown in Table 6.

The test results show that the proposed scheme cannot greatly reduce the time of model training when the domain name in the dataset is repeated. Based on the above characteristics, we can improve the structure of sketch or select multiple features as key in future research.

## 5. Conclusions and Future Work

In this paper, we present a novel APT attack detection approach based on the combination of the CK sketch structure and DNS traffic analysis. Our work allows security personnel to quickly identify and respond to potential APT attacks based on the analysis of DNS traffic. The proposed method leverages the unique characteristics of DNS traffic to identify potential APT attacks and utilizes the CK sketch structure to efficiently compress and summarize the traffic statistics. The experimental results demonstrate the effectiveness of the proposed approach in terms of both processing time and detection accuracy. Specifically, when applied to the ExtraTrees model, the processing time was reduced by approximately 50 times to 0.0638 s while maintaining a high accuracy of 0.97920. This reduction in processing time is expected to be achievable with other machine learning models as well.

It is important to note that the scope of our current work is limited to the communication control phase of APT attacks and does not take into account the characteristics of other phases. Additionally, the scheme may not provide significant time savings in cases where DNS traffic exhibits a high degree of variability in terms of domain names.

In future work, our focus will be on expanding the scope of APT attack characterization to encompass not only DNS traffic but also TCP and HTTP traffic. This will provide a more comprehensive understanding of APT attacks and allow for the selection of different attributes as keys in the sketch structure, providing greater adaptability to a wider range of APT attack scenarios. Additionally, we aim to gather more information about the suspect domain names and hosts involved in APT attacks for improved security analysis.

## Figures and Tables

**Table 1:** Comparison of existing APT detection methods.

Methods	Advantages	Disadvantages
Detection of unknown malicious traffic [33]	The unknown malicious samples showed high accuracy	Lack of real dataset
Detection of certain APT group identification [12]	Two features of original detection are proposed	The detection accuracy is not high
PCA or PCC combined with machine learning [32,34]	It can better extract the characteristics of APT attack	The number of datasets is small and the model training time is long
DNS traffic analysis [35,36,37]	High accuracy of model recognition	DNS characteristics of APT attacks need to be accurately determined

**Table 2:** Malicious dataset attributes.

Dataset	File Name	File Size
contagio	Sality	38.2 MB
	XTremeRAT	3.50 MB
	BIN_TrojanCookies	905 KB
	BIN_Sanny-Daws	868 KB
	BIN_Lagulon	626 KB
	PDF_CVE-2011-2462	341 KB
	8202_tbd	323 KB
	BIN_XtremeRat	308 KB
	BIN_LetsGo_yahoosb	306 KB
	BIN_8202	305 KB
	BIN_Taidoor	196 KB
ICEDID	2020-07-14-IcedID	3.72 MB
	2020-05-19-IcedID	3.60 MB
FORMBOOK	2020-07-09-Formbook	1.55 MB
Zloader	2020-06-09-ZLoader	6.18 MB

**Table 3:** Experimental dataset attributes.

Type	DNS Packet Num	Type of Filtered	Size of Data
Malware traffic	21,120	15	3.58 MB
Normal traffic	1,088,280	1	193.2 MB

**Table 4:** APT detection results.

	Precision	Recall	F-Score	Accurary	Training Duration/(s)	Testing Duration/(s)
LR	0.82774	0.77266	0.76728	0.77859	0.082741	0.069814
KNN	0.96523	0.96540	0.96463	0.96463	0.073833	0.348069
GaussianNB	0.78524	0.66480	0.63183	0.67442	0.005000	0.005950
DecisionTree	0.97055	0.97096	0.97044	0.97045	0.020942	0.025300
Bagging	0.96819	0.96802	0.96705	0.96705	0.142618	0.522602
RandomForest	0.96515	0.96472	0.96366	0.96366	0.042885	0.054854
ExtraTrees	0.97558	0.97592	0.97529	0.97529	0.063829	0.066821
AdaBoost	0.96374	0.96393	0.96318	0.96318	0.092753	0.088790

**Table 5:** Comparison with recent studies.

Scheme	Accuarcy	Recall	Training Duration/(s)
Scheme of this paper	0.97981	0.97981	0.0624
Yan Guanghua	0.96711	0.96787	3.1746

**Table 6:** Limitation contrast experiment.

Scheme	Training Duration/(s)
Sketch	0.0647
Non-sketch	0.0646

## Data Availability

In this paper, the malicious APT attacks DNS datasets are from two sites: https://www.netresec.com/ and http://contagiodump.blogspot.com/, which are accessed on 10 November 2022. The secure network DNS traffic cannot be shared due to privacy issues.

## Abbreviations

The following abbreviations are used in this manuscript:

APT	Advanced Persistent Threat
TP	True Positive
TPR	True Positive Rate
TN	True Negative
FP	False Positive
FN	False Negative
Iot	Internet of Things
CK	Cuckoo
TTL	Time To Live
LR	Logistic Regression
DNS	Domain Name Server
MDPI	Multidisciplinary Digital Publishing Institute
DOAJ	Directory of Open Access Journals
TLA	Three letter acronym
LD	Linear dichroism
